# Bio-hydrogen production by dark anaerobic fermentation of organic wastewater

**DOI:** 10.3389/fchem.2022.978907

**Published:** 2022-09-06

**Authors:** Xinghong Qu, Hongxue Zeng, Yongsheng Gao, Tiande Mo, Yu Li

**Affiliations:** ^1^ Zhejiang Tongji Vocational College of Science and Technology, Hangzhou, China; ^2^ Smart City Division, Hong Kong Productivity Council (HKPC), Hong Kong, China

**Keywords:** organic wastewater, hydrogen, anaerobic fermentation, biotechnology, product inhibition

## Abstract

Using organic wastewater to produce hydrogen by fermentation can generate clean energy while treating wastewater. At present, there are many inhibitory factors in the hydrogen production process, resulting in unsatisfactory hydrogen yield and hydrogen concentration during the fermentation process, and there are still great obstacles to the industrial promotion and commercial application of organic wastewater fermentation hydrogen production. This paper summarizes the hydrogen production of organic wastewater dark anaerobic fermentation technology. The current anaerobic fermentation hydrogen production systems and technologies are summarized and compared, and the factors and potential conditions that affect the performance of hydrogen production are discussed. The further requirements and research priorities for the market application of fermentation biohydrogen production technology in wastewater utilization are prospected.

## 1 Introduction

Energy consumption is steadily growing as the economy and society advance. In 2016, fossil fuels accounted for more than 80% of worldwide primary energy consumption. Although it has dropped from 81% in 2015, fossil fuels such as coal, oil and natural gas are still the main energy consumption (International Energy Agency, 2003). However, fossil fuels are unsustainable, and their combustion produces greenhouse gases and other pollutants. The development of green and clean renewable energy is an inevitable requirement for sustainable development (Ghosh and Mark, 2011). Hydrogen energy is known as the most promising clean energy in the twenty-first century because of its high calorific value, lack of greenhouse gas emissions, and lack of pollution from combustion. It helps to alleviate and avoid problems such as energy shortages, global warming, and environmental pollution (Lu et al., 2013).

Organic wastes (such as lignocellulose, organic waste, sewage sludge, organic wastewater, etc.) contain huge energy, and each kg of COD produces about 1.4 × 10^7^ kg of metabolic heat. These potential organic energy recovery and utilization have important practical significance (Zhang et al., 2018; Kumar and Samadder, 2020). Canadian scholars Shizas and Bagley pointed out (Shizas and Bagley, 2004) that the energy contained in sewage is 9.3 times of the energy consumed to treat them. If 10% of the energy can be used, it can satisfy the operation of the sewage treatment plant. As a result, extracting energy from organic waste is unquestionably crucial for the development of a low-carbon “energy saving and emission reduction” model as well as the development of renewable energy (Yi et al., 2018; Shen and Zhang, 2020; Ma et al., 2021). It is predicted that by 2070, 70% of the world’s energy will depend on renewable energy. Bioenergy technology mainly recovers bioenergy while processing organic pollutants through the action of microorganisms and their enzymes (Moya et al., 2017; Shen and Ma, 2022). It mainly includes biological fermentation methane production, biological hydrogen production, biological electricity production, etc. Among them, biological hydrogen production and electricity production have shown new attractiveness and strategic value due to their dual economic and ecological benefits.

Traditional hydrogen production methods use fossil fuels as raw materials (Lu et al., 2013), and use water electrolysis, thermochemistry, photochemistry, plasma chemistry and other methods to produce hydrogen, which consumes high energy and still needs to consume non-renewable energy. The biological hydrogen production technology of organic wastewater fermentation method utilizes the biological anaerobic-acid-generating fermentation process to produce hydrogen, and can also be used as the acid-generating phase in the two-phase anaerobic biological treatment system. Anaerobic fermentation hydrogen production technology has low energy consumption, simple process, high hydrogen conversion rate, and is conducive to waste recycling. It has attracted attention in the development and industrialization of hydrogen energy and has good prospects (Wang et al., 2021).

Biological hydrogen generation has not been widely employed to treat production and household wastewater due to cost and hydrogen production efficiency restrictions. There are more topics to be examined and studied, such as how to enhance hydrogen production efficiency, lower production costs, and increase substrate utilization (Akhlaghi and Najafpour-Darzi, 2020; Sivaramakrishnan et al., 2021). According to whether the input of external light energy is required, the biological hydrogen production technology mainly includes the hydrogen production of photo-splitting water, the hydrogen production of light fermentation, the hydrogen production of dark fermentation, and the coupled biological hydrogen production technology of dark-light fermentation. Among several hydrogen production methods, the fermentation biological hydrogen production technology has a large amount of hydrogen production and a fast hydrogen production rate, does not require input of light energy, and avoids the restriction of light energy factors. A wide range of solid or liquid waste biomass can be used as substrates, and the research and application are relatively mature. Using organic wastewater as a substrate can treat wastewater while generating energy. In addition, further research on dark fermentation will also contribute to the development of joint fermentation research. This review focuses on the development status of biological hydrogen production by anaerobic fermentation of organic wastewater and discusses the influencing factors of biological hydrogen production process. The future research directions are prospected by summarizing a series of problems faced by the technology towards maturity.

## 2 Theory of hydrogen production by fermentation of organic wastewater

**FIGURE 1:**
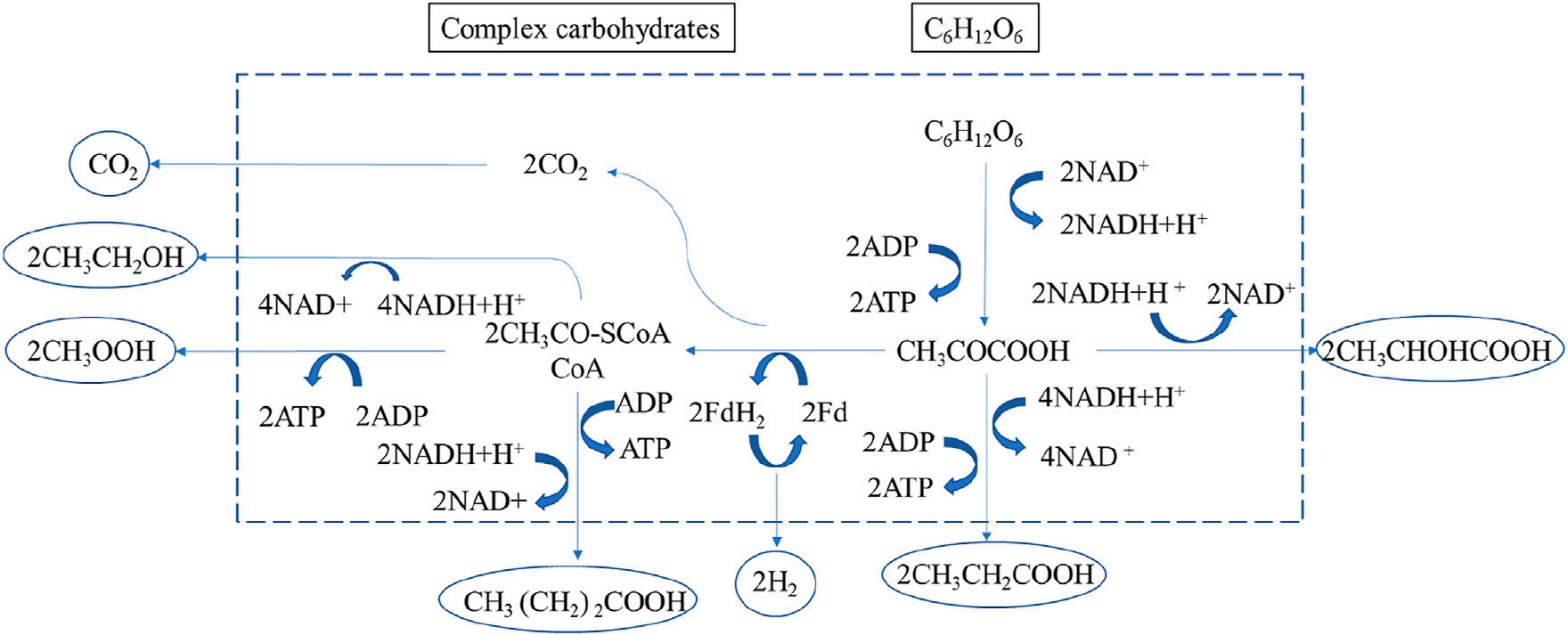
Carbohydrate fermentation pathways (Ren et al., 2007).

Carbohydrate-rich substrates are degraded anaerobically by hydrogen-producing microorganisms such as facultative anaerobes and obligate anaerobes in dark fermentation processes. The action of hydrogenase enzymes produces molecular hydrogen (H_2_) during the reduction of surplus electrons. Protons (H^+^) can operate as electron acceptors in an anaerobic environment, neutralizing electrons produced by the oxidation of organic substrates and creating H_2_. In contrast to aerobic respiration, water is the end result of anaerobic respiration (Karadag et al., 2014; Sivagurunathan et al., 2016). H_2_-producing bacteria convert glucose to pyruvate via the glycolytic pathway, which serves as a model substrate in the dark fermentation of glucose. It generates adenosine triphosphate (ATP) and a reduced form of reduced coenzyme I (NADH) from adenosine diphosphate (ADP). Pyruvate ferredoxin oxidoreductase and hydrogenase further convert pyruvate to acetyl-CoA, carbon dioxide (CO_2_), and H_2_. Pyruvate can also be converted to acetyl-CoA and formate, which can be further converted to H_2_ and CO_2_ depending on the kind of microorganism and ambient circumstances. Acetate, butyrate, and ethanol may all be produced from acetyl-CoA (Reverberi et al., 2016). Figure 1 shows a schematic diagram of the different steps and biochemical pathways of dark fermentation in complex carbohydrates by mixed anaerobic microorganisms, which can lead to a wide range of intermediates and by-products, depending on operating parameters such as substrate type, substrate loading rate, pH, temperature, and other operating and environmental conditions, as well as affecting microbial community structure in bioreactors. As the main body in the system, stringent anaerobic bacteria (*clostridium*, methylotrophic bacteria, rumen bacteria, methanogens, fungi, and so on) and facultative anaerobes (*Escherichia coli*) can carry out these metabolic steps (Usman et al., 2019; Rambabu et al., 2021).

Figure 1 shows the metabolic pathway of complex carbohydrates: monosaccharides are generated through water, pyruvate is generated through glycolysis, and after fermentation, it is converted into lactic acid, acetic acid, propionic acid, butyric acid, ethanol, etc. The end product is governed by the energy production process, the redox process of NADH/NAD^+^, and the number of acidic ends of the fermentation product. The standard Gibbs free energy change for glucose fermentation is shown in Table 1. In terms of Gibbs free energy (Hosseinzadeh et al., 2022), the conversion from glucose to acetic acid, propionic acid, butyric acid, ethanol or lactic acid can proceed spontaneously. However, under different microbial community structures and environmental conditions, the metabolic pathways of fermenting microorganisms and the conversion rates of each product are also different. For example, Guo et al. (2014) showed in studies with lignocellulosic substrates that the concentrations of the liquid phase end products acetic and butyric acids were always suboptimal, especially in batch experiments where homoacetogenic activity predominated. Therefore, the H_2_ yield is also lower at higher acetic acid concentrations. Certain homoacetogens belonging to the genus *Clostridium* can reduce H_2_ yield by converting H_2_ and CO_2_ to acetate or directly converting hexose to acetate (Kim et al., 2006a). In addition, the fermentation pathway can be inferred from the analysis of liquid end products.

**TABLE 1:** Standard Gibbs free energy change of glucose fermentation.

Reaction (pH = 7, T = 298.15 K)	$\Delta G^{0}\left(\text{kJ}/\text{mol}\right)$
${\text{C}}_{6}{\text{H}}_{\text{12}}{\text{O}}_{\text{6}}+4{\text{H}}_{\text{2}}\text{O}+2{\text{NAD}}^{+}\to 2{\text{CH}}_{\text{3}}{\text{COO}}^{-}+2{\text{HCO}}_{3}^{-}+2\text{NADH}+2{\text{H}}_{2}+6{\text{H}}^{+}$	−215.67
${\text{C}}_{\text{6}}{\text{H}}_{\text{12}}{\text{O}}_{\text{6}}+2\text{NADH}\to 2{\text{CH}}_{\text{3}}{\text{CH}}_{\text{2}}{\text{COO}}^{-}+2{\text{NAD}}^{+}+2{\text{H}}_{\text{2}}\text{O}$	−357.87
${\text{C}}_{\text{6}}{\text{H}}_{\text{12}}{\text{O}}_{\text{6}}+2{\text{H}}_{\text{2}}\text{O}\to 2{\text{CH}}_{\text{3}}{\text{CH}}_{\text{2}}{\text{CH}}_{\text{2}}{\text{COO}}^{-}+2{\text{HCO}}_{3}^{-}+2{\text{H}}_{2}+3{\text{H}}^{+}$	−261.46
${\text{C}}_{\text{6}}{\text{H}}_{\text{12}}{\text{O}}_{\text{6}}+2{\text{H}}_{\text{2}}\text{O}+2\text{NADH}\to 2{\text{CH}}_{\text{3}}{\text{CH}}_{\text{2}}\text{OH}+2{\text{HCO}}_{3}^{-}+2{\text{NAD}}^{+}+2{\text{H}}_{2}$	−234.83
${\text{C}}_{\text{6}}{\text{H}}_{\text{12}}{\text{O}}_{\text{6}}\to 2{\text{CH}}_{\text{3}}{\text{CHOHCOO}}^{-}+2{\text{H}}^{+}$	−217.70

Fermentation types in the anaerobic hydrogen generation system may be separated into ethanol-type fermentation, butyric acid-type fermentation, and propionic acid-type fermentation based on the composition of the terminal fermentation products (Wang et al., 2020).

### 2.1 Ethanol-type fermentation

The end products of ethanolic fermentation are ethanol, acetic acid, H_2_, CO_2_ and a small amount of butyric acid. The ethanol type is mainly carried out under the action of *Bacteroides*, *Pseudomonas fermentum* and *Fusobacterium*, and *Bacteroides* and *Fusobacterium* are absolutely dominant. Compared with the propionic acid-type fermentation pathway, bacterial ethanol fermentation has the same oxidative capacity for NADH + H^+^, and each 1 mol of glucose oxidized can regenerate 4 mol of NAD^+^, while the butyrate-producing pathway can only regenerate 2 mol of NAD^+^. Therefore, ethanol can regenerate 4 mol of NAD^+^. The reaction coupled with acetic acid has a strong ability to adjust the balance of NADH + H^+^/NAD^+^, and ethanol-type fermentation has stronger stability and higher hydrogen production capacity than butyrate-type fermentation (Li et al., 2009; Li et al., 2013).

### 2.2 Butyric acid fermentation

*Clostridium* bacteria ferment glucose and generate pyruvate through the EMP pathway. Pyruvate is catalyzed by pyruvate ferredoxin oxidoreductase to generate acetyl-CoA, and acetyl-CoA is finally converted to butyrate through a series of transformations. Pyruvate is catalyzed by pyruvate ferredoxin oxidoreductase to generate acetyl phosphate and release H_2_ and CO_2_. Butyric acid, acetic acid, H_2_, CO_2_, and a little quantity of propionic acid are the principal end products of butyric acid fermentation. *Clostridium* is the most common organism that causes butyric acid fermentation (Dessì et al., 2020; Chen et al., 2021a).

### 2.3 Propionic acid fermentation

Propionic acid and acetic acid, which release relatively little gas, are the primary fermentation end-products of propionic acid fermentation. Propionic acid fermentation is mostly carried out by Propionibacterium, a bacteria that lacks hydrogenase and so does not produce hydrogen (Ali et al., 2021).

The core of oxygen fermentation hydrogen production technology is anaerobic hydrogen-producing microorganisms. Among the anaerobic hydrogen-producing microorganisms, obligate anaerobic bacteria include *Clostridium*, Methylotrophs, Methanogenic bacteria, Rumen bacteria and some archaea etc. These bacteria do not contain a cytochrome system and produce hydrogen through a metabolic pathway that produces pyruvate or pyruvate (Chen et al., 2021b).

Facultative anaerobes, including *Escherichia coli* and *Enterobacter*, contain a cytochrome system and produce hydrogen through their own metabolic pathway for decomposing formic acid (Gray and Gest, 1965). At present, most studies on fermentative hydrogen production focus on *Clostridium* and *Enterobacter* (Vasconcelos et al., 2016; Pugazhendhi et al., 2019), whose main metabolites are acetate and butyrate. There are also differences in the hydrogen production efficiency of different bacterial groups using the same substrate. Strict anaerobes generally have higher hydrogen production capacity than facultative anaerobes. The types of carbohydrate fermentation and their main end products and typical microorganisms are shown in Table 2.

**TABLE 2:** Main classical types of carbohydrate fermentation.

Fermentation type	Main end product	Typical microorganism
Butyric acid fermentation	Butyric acid; acetic acid; H_2_ + CO_2_	*Clostridium*; *C. butyricum*; *Butyrivibrio*
Propionic acid fermentation	Propionic acid; acetic acid, CO_2_	*Propionibacterium*; *Veillonella*
Mixed acid fermentation	Lactic acid; acetic acid; ethanol; formic acid; H_2_+CO_2_	*Escherichia*; *Proteus*; *Shigella*; *Salmonella*
Lactic acid fermentation-isotype	Lactic acid	*Lactobacillus*; *Streprococcus*
Lactic acid fermentation-shaped	Lactic acid; ethanol; CO_2_	*Leuconostoc*
Ethanol fermentation	Ethanol; CO_2_	*Saccharomyces*; *Zymomonas*

## 3 Research status of organic wastewater fermentation hydrogen production process

Existing studies have studied hydrogen production in a variety of reactors, among which the most studied include (de Menezes and Silva, 2019) Continuous flow Stirred Tank Reactor (CSTR), Upflow Anaerobic Sludge Bed (UASB), Anaerobic Baffled Reactor (ABR), Expanded Granular Sludge Bed (EGSB), membrane reactors, etc.

### 3.1 Continuous flow stirred tank reactor

The continuous flow stirred tank reactor (CSTR) has an integrated reaction zone and precipitation zone, as well as a gas-liquid-solid three-phase separation device and a stirring device. Organic wastewater or solid waste is used as fermentation substrate, and is pumped into the reactor at a certain flow rate from the water inlet at the bottom of the reactor according to the hydraulic retention time. The inside of the reactor is in a state of complete stirring, and the substrate and the mud-water mixture in the reactor are rapidly homogeneous. However, Zhang et al. (2021) developed and successfully controlled and operated a pilot-scale CSTR fermentation hydrogen production equipment in 1997, with an effective volume of 1.45 m^3^ and a continuous hydrogen production capacity of 30 mol/kg VSS d. When the temperature is 35°C, pH = 4.0–4.5, HRT = 4–6 h, ORP = −10.0 ∼ −12.5 mV, and volume load is 35–55 kg COD/m^3^ d, etc., the maximum continuous hydrogen production capacity of the reactor Up to 5.7 m^3^/m^3^ d. It has good resistance to load shock and stable operation. The COD removal rate can reach more than 20%, and the gas production rate can reach 26 mol/kgCOD. From 2002 to 2005, the production test was carried out on the basis of the pilot test and achieved success. The effective volume of the CSTR reactor was 63.5 m^3^, the hydrogen production capacity reached 4.57 m^3^/m^3^ d, and after bio-enhanced, it reached 5.32 m^3^/m^3^ d.

**FIGURE 2:**
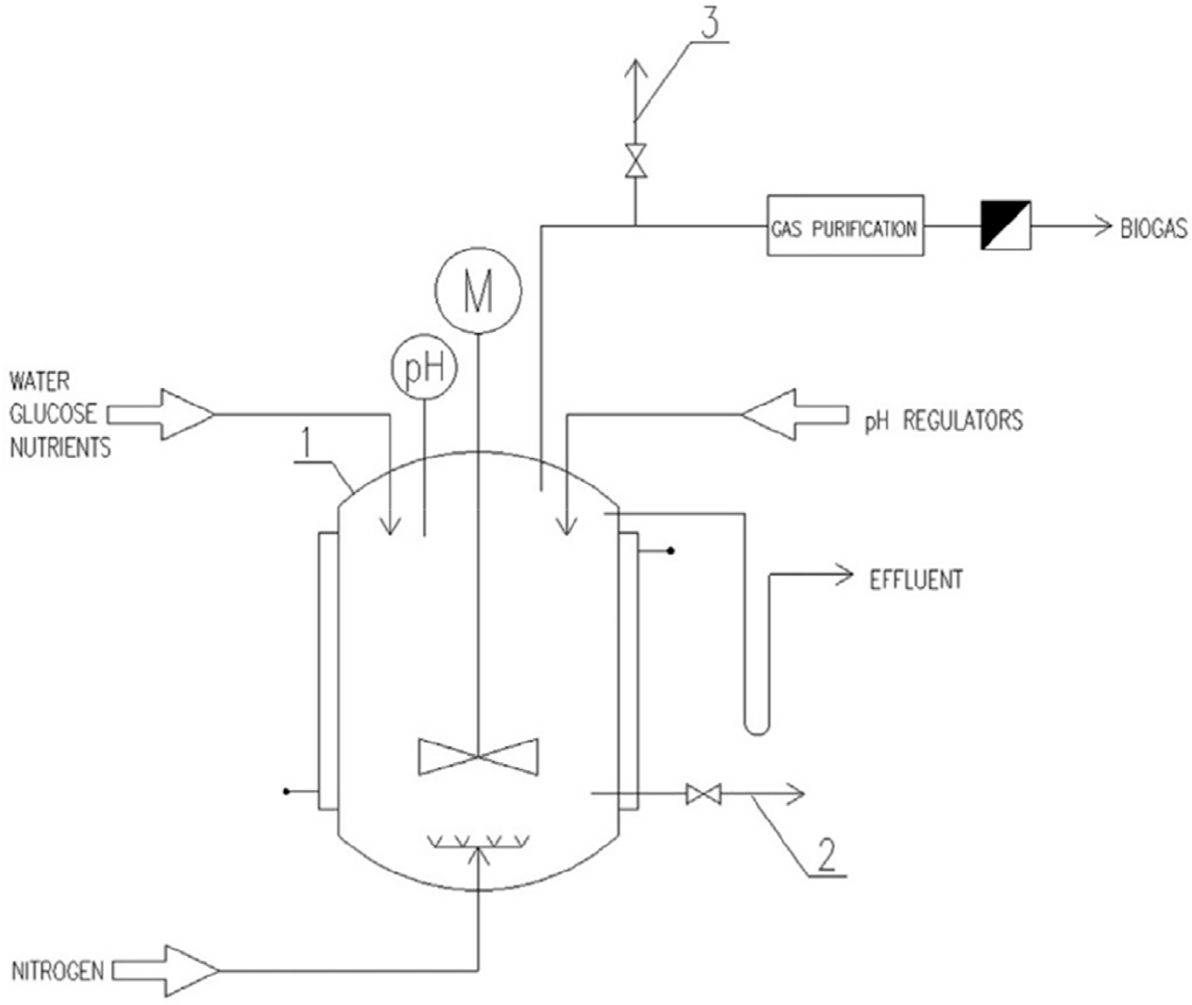
Schematic diagram of the CSTR reactor (Łukajtis et al., 2018).

As shown in Figure 2, the CSTR reactor substrate can be rapidly mixed with the microorganisms. The mixed liquid maintains a strong turbulent state, the mass transfer effect between the biomass and the substrate is good, and it is not easy to form granular sludge (the inside of the granular sludge or the deep layer of the biofilm will breed methanogens). Therefore, the operating conditions of the CSTR reactor can effectively inhibit the influence of methanogens on the hydrogen production process, and control the fermentation in the hydrogen production section. CSTR is more suitable for hydrogen production section of two-phase fermentation system based on phase separation and large-scale organic wastewater fermentation hydrogen production.

### 3.2 Upflow anaerobic sludge bed

**FIGURE 3:**
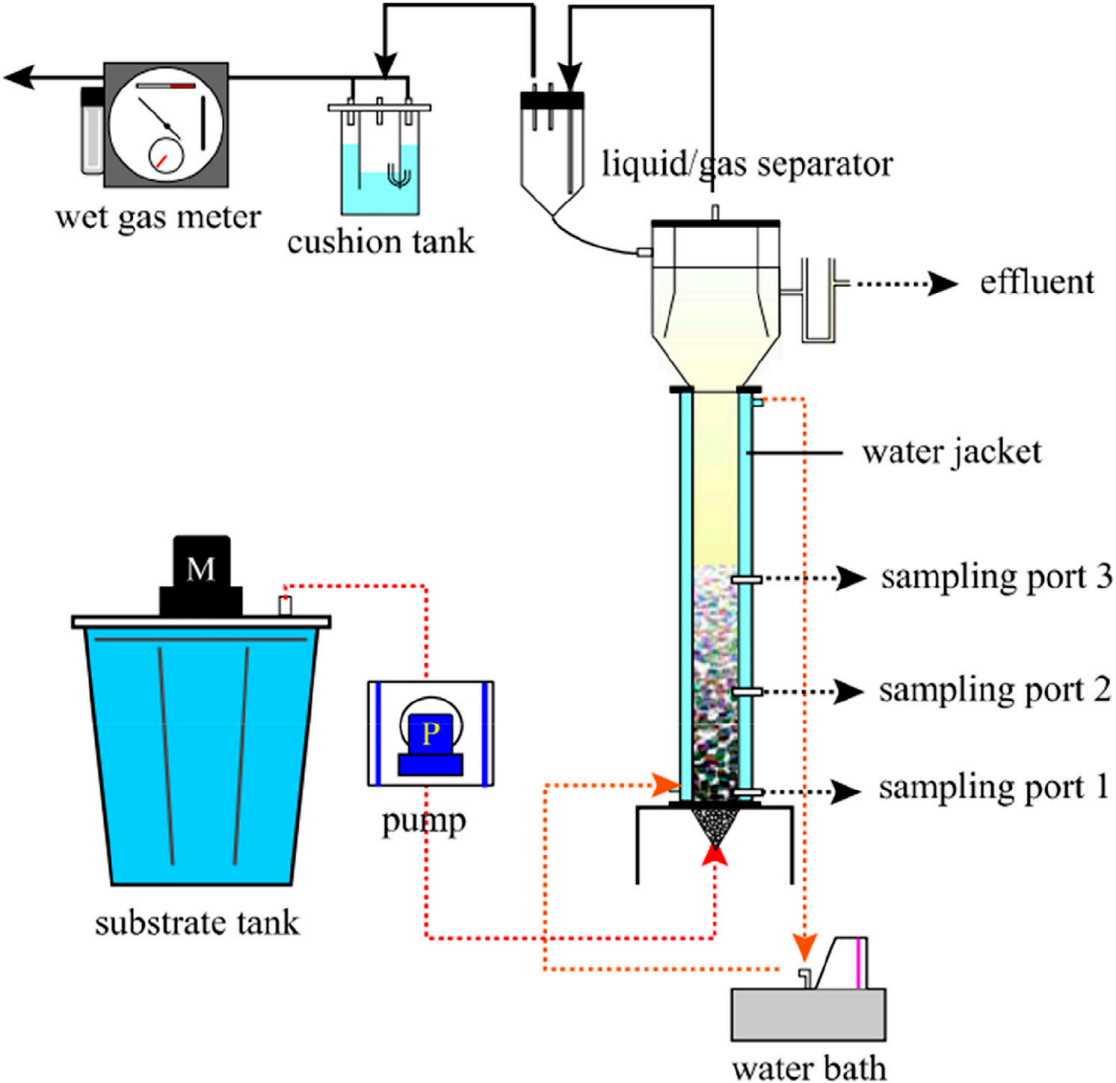
Schematic diagram of the UASB reactor (Akutsu et al., 2009).

The Upflow Anaerobic Sludge Bed (UASB) consists of a sludge zone, a suspended layer zone and a three-phase separator, as shown in Figure 3. The USAB reactor sludge has a longer residence time and can form a granular sludge, thus maintaining a high biomass. But it is also possible to grow methanogenic bacteria inside the granular sludge or the carrier to consume hydrogen to produce methane and reduce the hydrogen production.

Kotsopoulos et al. (2006) loaded hydrogen-producing sludge with UASB anaerobic granular sludge, and used UASB reactor to ferment hydrogen at high temperature (70°C), achieving a maximum hydrogen yield of 2.47 mol H_2_/mol lactose. The literature mentions that the reflux of the reactor mixture is beneficial to enrich the hydrogen-producing microorganisms to improve the hydrogen yield. Akutsu et al. (2009) used starch as a substrate to produce hydrogen with UASB, and the hydrogen production rate was only 0.08 L/Lh, which may be due to the long sludge residence time or the small organic load. Yongfeng et al. (2016) used UASB to treat molasses wastewater. The influent COD was 4,000 mg/L, HRT = 8 h, and the concentrations of ethanol and acetic acid were 840.56 mg/L and 403.12 mg/L respectively after 65 days of operation. The concentration accounted for 93.2% of the total concentration, and the hydrogen production performance of the system was the best. The hydrogen production rate was 2.079 mmol/Lh, and the hydrogen production rate was 1.12 m^3^/m^3^ d.

### 3.3 Anaerobic baffled reactor

ABR belongs to the third generation of anaerobic reactors derived from the SMPA (staged multi-phase anaerobic reactor) theory (Lettinga et al., 1997). A baffle plate is arranged in the reactor, and the waste water flows up and down along the baffle plate in the reactor, and passes through each compartment in sequence until the outlet. The ABR reactor has good sludge retention performance and hydraulic flow pattern, and has the characteristics of biological phase separation and good microbial functional division. Different microbial communities can be developed in each compartment. Li et al. (2017) started the ABR reactor to treat the simulated livestock and poultry breeding wastewater within 64 days, the OLR = 5.7 kg COD/m^3^ d, the average COD removal rate could reach 98%, and the granular sludge concentration was between 7.14 and 26.17 g/L. The results of PCR-DGGE analysis showed that the system contained propionic acid-producing bacteria and butyric acid-type hydrogen-producing bacteria. Guochen et al. (2013) used an ABR reactor at 35°C, under the condition of influent COD = 5,000 mg/L, the system achieved ethanol-type fermentation for 26 days, and the hydrogen yield was 0.13 L/g COD. The separation of the biological phase in the ABR reactor enables the organic matter to be used in a cascade and removed in an orderly manner to achieve the purpose of deep hydrogen production.

### 3.4 Anaerobic biofilm reactor

Anaerobic biofilm reactors rely on the formation of biofilms on the surface of fillers to produce hydrogen by fermentation. The most commonly used anaerobic biofilm reactors are anaerobic packed bed reactors (APBR) and anaerobic fluidized bed reactors (AFBR) (Barca et al., 2015). The biofilm reactor can overcome the problem that the hydraulic retention time of the suspension bioreactor is the same as the sludge retention time, and the sludge is easy to lose. The biofilm reactor sludge residence time is separated from the hydraulic residence time, so that higher biomass can be maintained and the hydrogen production effect can be improved. Perna et al. (2013) used APBR to treat cheese whey wastewater. The residence time of the mixed solution was 24 h, the organic load was 22–37 kg COD/m^3^ d, the hydrogen production capacity reached 1 m^3^/m^3^ d, and the reactor continued to operate stably, there was no clogging of the reactor, and no methane was detected in the fermentation gas. Zhang et al. (2007) used an AFBR reactor to treat simulated wastewater containing 10 g/L glucose for hydrogen production, and the maximum hydrogen production rate was 4.34 mmol/gVSS h and 2.36 L/L h, and the main liquid end product was propionic acid and butyric acid. Table 3 lists the operating parameters and results of several fermentative biological hydrogen production reactors.

**TABLE 3:** Operating parameters and hydrogen production performance of the fermentative biological hydrogen production reactor.

Reactor type	Underlying object	HRT/h	pH	Temperature/°C	Maximum hydrogen production	Maximum hydrogen production rate	References
CSTR	30 g COD/L Sucrose	12	5.4	35	1.22 mol/mol hexose	3.80^a^	Yang et al. (2012)
	20 g COD/L Sucrose, pour CO_2_ into the reactor	12	5.3	35	1.68 mol/mol hexose	6.89^a^	Nanqi et al. (2010)
	7.0 kg/(m^3^ d) Honey Wastewater	6	5	35	1.3 m^3^/(m^3^ d)	-	Muhamad et al. (2011)
	Glucose Artificial Sewage	24	5.5	37	1.0 mol/mol Glucose	132.2^b^	Mengjia et al. (2014)
	35–55 kg COD/m^3^ d Honey Wastewater	4–6	4.0–4.5	35	26 mol/kg·COD	5.7^c^	Zhang et al. (2007)
	Alcohol Wastewater	96	7.0	70	172.0 ml/g VSS	-	Ren et al. (2006)
UASB	10 g COD/L sucrose	13	7.0	39	1.16 mol/mol Glucose	144^b^	Chairattanamanokorn et al. (2009)
	10 g/L Glucose Artificial Sewage	8	5.5	37	1.93 mol/mol Glucose	6.92^c^	Feng et al. (2010)
ABR	Simulated Livestock Breeding wastewater	24	7–8	32	—	—	Li et al. (2017)
	Honey Wastewater	35	4.5	35	0.13 L/g COD	1.42^c^	Guochen et al. (2013)
AFBR	Glucose Simulated Wastewater	4	4.0	37	1.16 mol/mol Glucose	4.34^d^	Zhang et al. (2007)
APBR	Cheese Whey Wastewater	24	>5	30	0.668 mol/mol Lactic acid	1^c^	Perna et al. (2013)

Maximum hydrogen production rate unit.

a L/(gVSS d).

b ml/(L h).

c m^3^/m^3^ d.

d mmol/gVSS h.

It can be seen from Table 3 that different hydrogen production reactors have different operating parameters and hydrogen production efficiencies, and the hydrogen production efficiencies vary with the changes of substrate types, organic loads, pH, temperature and other factors. Factors including temperature, pH, nutrients, residence time, hydrogen partial pressure, etc. are all influencing factors in the process of hydrogen production by fermentation, and many scholars have studied these influencing factors (Perna et al., 2013).

## 4 Factors affecting biological hydrogen production by fermentation

The biochemical reactions that occur in the anaerobic fermentation of hydrogen production may vary according to its operational control factors, so the production and yield of hydrogen will be quite different. The metabolic pathway of anaerobic hydrogen production is affected by many factors. Factors such as temperature, pH, nutrients, inoculum and enrichment conditions, residence time, and hydrogen partial pressure will all affect the mechanism of anaerobic hydrogen production. Under different operating conditions and influent water quality conditions, the amount of hydrogen produced by anaerobic fermentation and the rate of hydrogen production will be quite different, and the biochemical reactions in the system will also be different. To improve the activity of hydrogen-producing bacteria in the anaerobic fermentation hydrogen production system and the hydrogen production performance of the system, there have been many studies on environmental factors. However, excessive accumulation of nutrient elements, metal ions, liquid end products, hydrogen partial pressure and other fermentation products will inhibit microorganisms. The influencing factors and possible inhibition of biological hydrogen production by fermentation are as follows.

### 4.1 Impact factor

The biochemical reactions that occur in the anaerobic fermentation of hydrogen production may vary according to its operational control factors, so the production and yield of hydrogen will be quite different. The metabolic pathway of anaerobic hydrogen production is affected by many factors. Factors such as inoculum and enrichment conditions, temperature, pH, nutrient salt, residence time, hydrogen partial pressure and other factors will affect the process of anaerobic fermentation hydrogen production mechanism. To improve the activity of hydrogen-producing bacteria in the anaerobic fermentation hydrogen production system and the hydrogen production performance of the system, there have been many studies on environmental factors.

#### 4.1.1 Temperature

**FIGURE 4:**
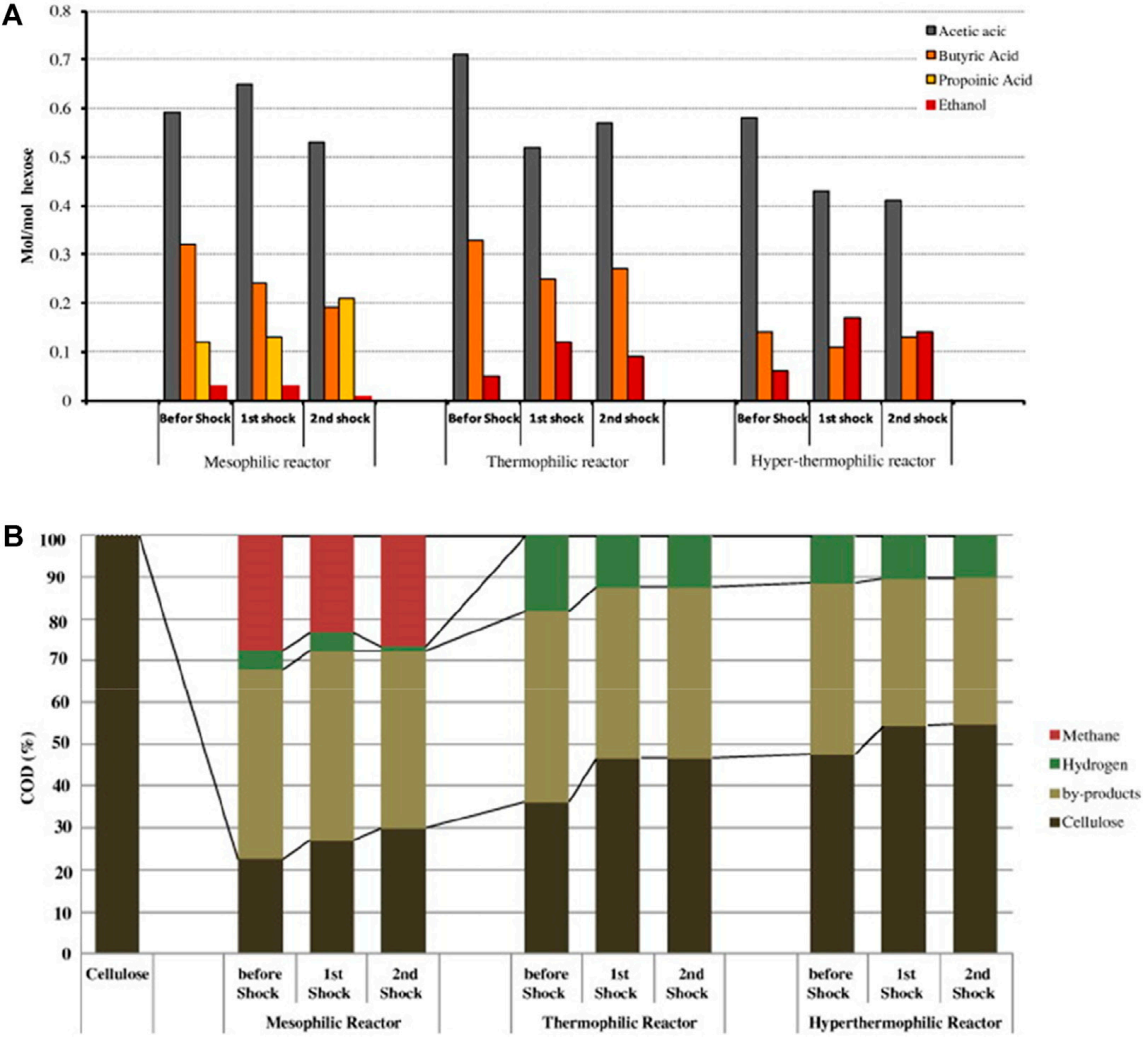
Effects of temperature and temperature shock on by-product production **(A)** and COD mass balance **(B)** (Gadow et al., 2013).

Temperature has a great influence on the biochemical reaction of hydrogen-producing bacteria. It is reported in the literature that the optimum temperature for most hydrogen-producing bacteria is 20–45°C, because most of the hydrogen-producing bacteria are mesophilic bacteria. The research of Mu et al. (2006) showed that between 33 and 39°C, the hydrogen yield increased with the increase of temperature, and after 39°C, the hydrogen yield decreased. When the temperature is controlled in the range of 35–38°C, the anaerobic activated sludge in the reactor has the most vigorous metabolism, and the gas production rate and hydrogen production rate are also the largest. It has been reported that high temperature is beneficial to anaerobic fermentation for hydrogen production, because high temperature can reduce the solubility of H_2_ in the liquid phase (Feng et al., 2010), and thermophiles in fermenting microorganisms have a higher tolerance limit to high temperature. But high temperature fermentation requires more thermal energy input, which will provide energy costs. Therefore, the fermentation temperature should be controlled at (36 ± 1)°C in combination with the actual wastewater and substrate conditions. As shown in Figures 4A,B, fermentation microorganisms are very sensitive to temperature changes, and the impact of temperature changes will reduce the biomass and hydrogen production rate in the reaction system, and it is difficult to recover after the temperature is adjusted back to the operating temperature (Gadow et al., 2013). Therefore, the daily temperature fluctuation of the anaerobic fermentation reactor should be controlled within 2–3°C. And it is necessary to pay more attention to the stable control of temperature during high-load operation, because the sensitivity of fermentation microorganisms to temperature changes is positively correlated with organic load.

#### 4.1.2 pH

To ensure the growth of hydrogen-producing bacteria and the action of hydrogen-producing enzymes, a suitable pH is very important. pH not only affects the hydrogen yield, but also affects the fermentation metabolic pathways, leading to different types of hydrogen-producing fermentations. The results of Ren et al. (2006) showed that ethanolic fermentation, mixed acid fermentation and butyric acid fermentation occurred at pH 4.5–4.7, 5.0–6.2, and 6.2–6.5, respectively. The kind of system fermentation is determined by the dominating bacteria in the hydrogen production system, but the ecological niches of ethanol-type, butyric-acid-type, and propionic-acid-type fermentation bacteria groups differ. As a result, the mixed bacteria in the reactor can go through a regular, directed, and predictable community succession process by keeping the pH value within a particular range. It can make the target flora dominate to become the top community, and obtain the type of fermentation that you want to maintain (Sheng and Feng, 2016).

#### 4.1.3 Nutrients

In the biological hydrogen production system, carbon, nitrogen, phosphorus and some inorganic metal elements are all nutrients required by hydrogen-producing bacteria. The carbon source generally comes from the fermentation substrate. The dark fermentation hydrogen production can utilize a wide range of substrates, and can utilize a variety of organic wastewater and solid organic matter. Nitrogen sources are abundant, and can be provided by protein, nitrate, nitrite, ammonium salt, etc. The presence of ammonium not only provides a nitrogen source but also acts as a buffer for organic acids (Wang et al., 2009). Phosphorus is generally provided by phosphates.

Metal ions are very important for the growth of hydrogen-producing bacteria and the synthesis of enzymes. Lin and Lay (2005) investigated trace amounts of Mg, Fe, Na, Zn, K, I, Co., NH^4+^, Mn, Ni, Cu, Mo and Ca. Effects of elements on the hydrogen production performance of mixed flora with C pasteurianum as the dominant flora. The results show that Mg, Fe, Na, and Zn are crucial for hydrogen production. When the medium composition contained 120 mg/L MgCl_2_·2H_2_O, 1000 mg/L NaCl, 0.5 mg/L ZnCl_2_ and 3 mg/L FeSO_4_·7H_2_O, the hydrogen production capacity of the mixed flora was the best. Fe is an important part of hydrogenase, and the production and consumption of hydrogen are all completed under the action of hydrogenase. Iron affects the synthesis of formate lyase and then the process of formate decomposition to produce hydrogen, and Fe is a major component of ferredoxin that catalyzes the production of hydrogen (Shima et al., 2008). The lack of Ni will affect the growth of anaerobic fermentation bacteria, and the hydrogen production efficiency is significantly improved when the concentration of Ni is appropriately increased (Trchounian et al., 2017). However, excess nutrients and metal ions will inhibit microorganisms.

### 4.2 Possible inhibition

#### 4.2.1 Mutual inhibition between bacterial species

Hydrogen production technology can be divided into pure culture fermentation hydrogen production and mixed bacterial group fermentation hydrogen production according to whether a single bacteria or a variety of bacteria are used. Although the pure culture fermentation hydrogen production system can achieve a high hydrogen yield (Yang and Wang, 2019; Shen, 2021), the microbial diversity of the mixed flora is conducive to the hydrolysis and transformation of the substrate, hydrogen production, and can also improve the stability and sustainability of the system. Moreover, hydrogen production by mixed bacterial flora fermentation is more feasible from both economic and engineering perspectives. Therefore, in this study, mixed bacterial flora was used to produce hydrogen by fermentation. However, there are limitations in hydrogen production by mixed bacterial flora fermentation—there may be hydrogen-consuming bacteria (HCB) or microorganisms that compete with hydrogen-producing bacteria in the mixed bacterial flora, resulting in a decrease in net hydrogen production and a decrease in the rate of hydrogen production.

Microorganisms that inhibit or compete with the hydrogen production process include hydrogen-consuming methanogens, homoacetogens (Saady, 2013), sulfate-reducing bacteria, propionic acid-producing bacteria, nitrate-reducing bacteria, iron-reducing bacteria, and lactic acid bacteria. Homoacetogens, propionogens can use hydrogen as an electron donor for the production of propionic acid from glucose (Luo et al., 2010). Propionate is also produced by propionate-producing bacteria such as *Clostridium propionicum* and *Clostridium homopropionicum* when lactic acid is degraded. The process consumes NADH, which has a detrimental impact on biological hydrogen synthesis (Saady, 2013). It has been reported that propionic acid has an inhibitory effect on hydrogen production by biological fermentation (Chairattanamanokorn et al., 2009). Propionate inhibits the dark fermentation process through the production of propionate, in addition to causing a decrease in hydrogen yield through direct consumption of hydrogen or through utilization of NADH.

#### 4.2.2 Metal ion suppression

**FIGURE 5:**
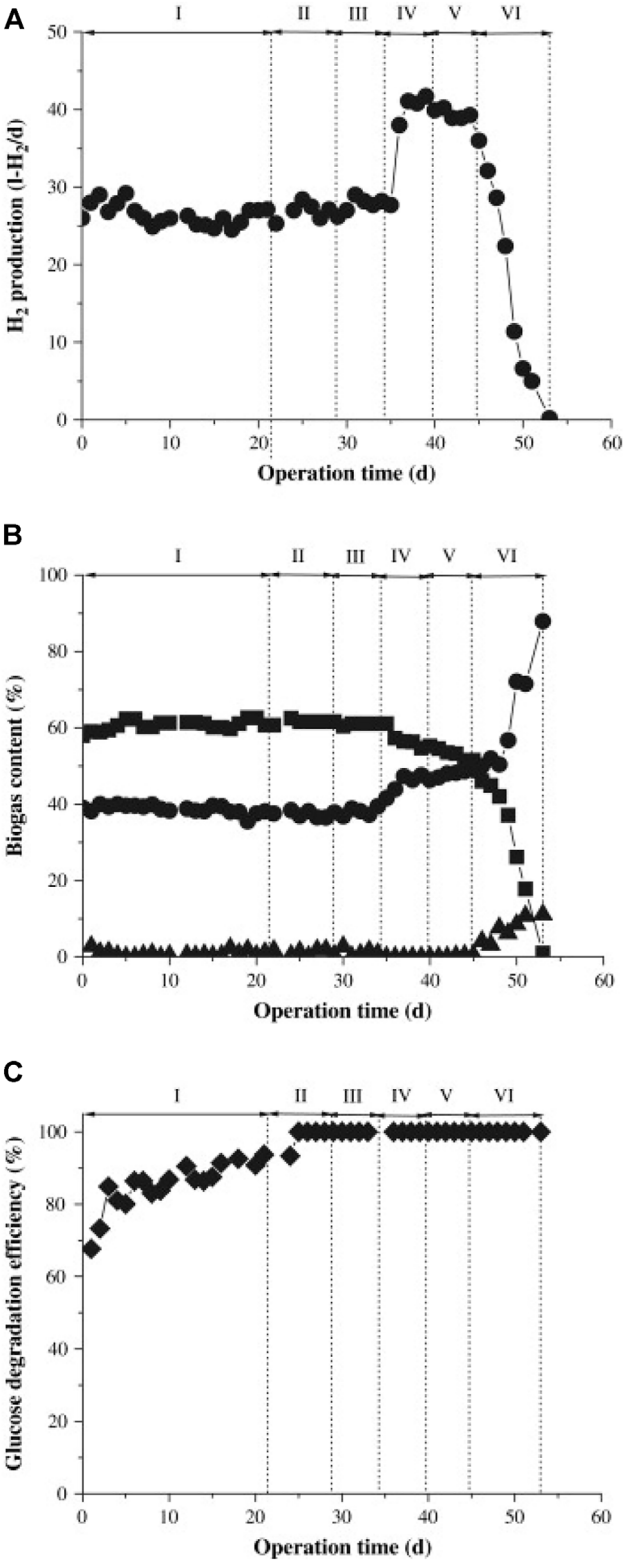
Changes in H_2_ production, biogas content, and glucose degradation efficiency with addition of FeSO_4_ concentration. Symbols: **(A)** (●), H_2_ production; **(B)** (▪), H2; (●), CO_2_; (▴), N_2_ in biogas content; **(C)** (⋄); glucose degradation efficiency (Lee et al., 2009).

Metal ions are an important condition for the dark fermentation hydrogen production process because they contribute to bacterial metabolism, cell growth, enzyme and coenzyme activation and function and biological hydrogen production (Wang and Wan, 2009). However, high concentrations of metal ions can prove to inhibit the hydrogen-producing process and hydrogen-producing microorganisms. Metal ions may be present in the inoculum or substrate, including light metal ions such as magnesium ions, sodium ions, and calcium ions, or heavy metal ions such as iron ions, nickel ions, copper ions, and zinc ions. Yongfeng et al. (2013a) studied the hydrogen production efficiency of Biohydrogenbacterium R3 sp.nov under the influence of different concentrations of metal ions. It was reported that when the CoCl_2_ concentration was in the range of 1.00–2.00 mg/L, the hydrogen production of the system was equal to the cell concentration began to decline, resulting in the phenomenon of high concentration inhibition. Ferrous ions are also implicated in the gene expression of important enzymes involved in hydrogen generation metabolism (Yongfeng et al., 2013b), and low ferrous ion concentrations can stimulate hydrogen production. Iron is required for the development and metabolism of hydrogen-producing bacteria and has an impact on hydrogenase structure and activity. However, high concentrations of ferrous ions still inhibit the hydrogen production process. Lee et al. (2009) reported that the concentration threshold of ferrous ions was 10.9 mg/L. As shown in Figures 5A–C, after the threshold was exceeded, the hydrogenase activity decreased and the hydrogen production rate decreased.

#### 4.2.3 Inhibition by matrix pretreatment

In dark fermentation anaerobic hydrogen production reactors, pretreatment can be applied to inoculum and substrate. Inoculation pretreatment was used to enrich hydrogen-producing bacteria, inhibit hydrogen-consuming bacteria and other competing bacteria (Xia and Ruying, 2017). Matrix pretreatment is used to release cellulose molecules into solution when using materials such as lignocellulose as substrates, breaking the crystalline structure of cellulose and assisting depolymerization to enhance substrate hydrolysis and facilitate biological hydrogen production (Lingling, 2014). In addition to these positive aspects, preconditioning may also lead to the formation of inhibition. Toxic by-products such as phenolic compounds, furan derivatives and weak acids, which inhibit the production of hydrogen by dark fermentation organisms (Palmqvist and Hahn-Hägerdal, 2000; Chen et al., 2013; Behera et al., 2014).

#### 4.2.4 Inhibition of soluble fermentation product accumulation

The hydrogen generation process, as well as other side reactions, will yield soluble metabolites when the dark fermentation biological hydrogen production system produces hydrogen. Organic acids such as acetic, propionic, butyric, formic, and lactic acids, as well as alcohols and ketones such as ethanol, acetone, and butanol, are examples. The synthesis of these metabolites is separated into acid-producing and alcohol-producing kinds, depending on the microbial population and metabolic pathways (Wong et al., 2014). Metabolic pathways depend on many factors such as inoculum type, pretreatment technique, substrate type and concentration, pH (Siddiqui et al., 2017), temperature and H_2_ partial pressure. Although low end product concentrations may be advantageous for fermentative hydrogen generation methods. High concentrations, on the other hand, may cause process inhibition, resulting in lower hydrogen generation, a lower hydrogen production rate, and the buildup of inhibition. Both dissociated and undissociated forms of the soluble end products exist in the system, and both limit biological hydrogen production (Li et al., 2012). Free acids raise the ionic strength of the medium, which can cause HPB cells to lyse, reducing hydrogen generation. Undissociated acids can infiltrate cells and subsequently dissociate owing to greater intracellular pH, raising intracellular hydrogen ion concentration and generating pH imbalances that might disrupt metabolic activity and other metabolic functions, leading to cellular death (Palmqvist and Hahn-Hägerdal, 2000). According to Wang et al. (2008), acetic acid has a larger inhibitory impact than ethanol, while ethanol has a lower inhibitory effect than acetic acid, butyric acid, or propionic acid.

#### 4.2.5 Inhibition of hydrogen partial pressure generation

Hydrogen partial pressure is a critical element in dark fermentation organisms’ hydrogen synthesis, and hydrogen-producing bacteria are extremely sensitive to hydrogen partial pressure. The partial pressure of hydrogen, which can be properly controlled to enhance the effect of hydrogen production by anaerobic fermentation with either solid or liquid substrates, is a factor worth studying in the process of hydrogen production by anaerobic fermentation with either solid or liquid substrates (Levin et al., 2004). According to Henry’s law, the dissolved hydrogen concentration in the reaction liquid is affected by the hydrogen partial pressure in the gas phase. The hydrogen synthesis process is inhibited and the biological hydrogen production impact is reduced when the hydrogen concentration in the liquid phase rises. The dark fermentation process mainly produces hydrogen through the reduction of protons by ferritin or reduced coenzyme. From a chemical thermodynamic point of view, a high concentration of hydrogen partial pressure is unfavorable for the reduction of protons. This causes the oxidation of hydrogen to occur more easily, resulting in lower hydrogen yields.

**FIGURE 6:**
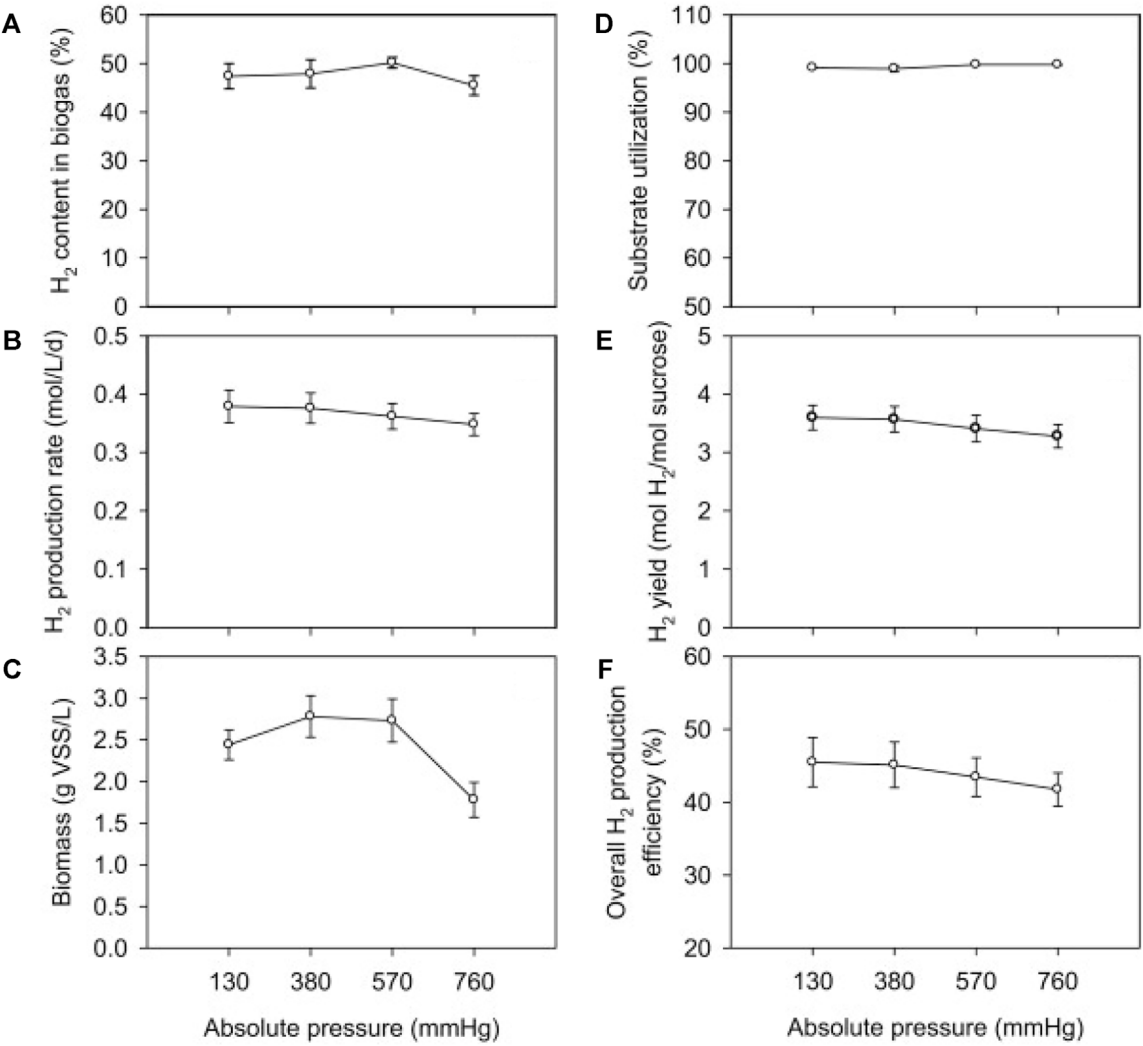
The effect of pressure on **(A)** H_2_ content in biogas, **(B)** H_2_ production rate (HPR), **(C)** biomass concentration, **(D)** substrate utilization, **(E)** H_2_ yield (HY), and **(F)** overall H_2_ production efficiency when dark fermentation was operated under the condition of: HRT, 12 h; temperature, 37°C; substrate (sucrose) concentration, 17.8 g/L (Lee et al., 2012).

Therefore, some studies have reported methods to reduce the inhibition of hydrogen partial pressure. Chang et al. (2012) compared the hydrogen production under continuous and intermittent release of fermentation gas. It was found that the continuous release of the fermentation gas resulted in a higher hydrogen production rate, and in addition, providing lye to absorb the carbon dioxide in the released gas could further release the gas and strengthen the hydrogen production process. Lee et al. (2012) used sucrose as the main carbon source, inoculated coastal sludge, and conducted a fermentation hydrogen production experiment in a CSTR reactor. As shown in Figures 6A–F, it was found that reducing the pressure increased the reaction efficiency and thus significantly increased the hydrogen yield, reaching 4.50 mol H_2_/mol sucrose at a HRT of 6 h. At 380 mmHg, the hydrogen yield increased by about 8% compared to 760 mmHg. And reducing the pressure can have a better effect on the system with low residence time and high hydrogen production rate. Nguyen et al. (2010) adopted the method of nitrogen stripping, and in the fermentation hydrogen production system with glucose as the substrate, the hydrogen production rate increased by 78% compared with the control group. The rate of hydrogen synthesis in a hydrogen production system employing xylose as a substrate rose by 56%. The strategy of raising headspace capacity was also employed to lower hydrogen partial pressure in the same investigation. The greatest gas output for biological hydrogen generation is achieved when the headspace volume to liquid volume ratio in the reactor is 2:1.

The main methods for reducing the hydrogen partial pressure in the hydrogen production system are to accelerate the stirring speed in the reactor to reduce the dissolved hydrogen concentration in the solution, to aerate the reactor with an inert gas to reduce the hydrogen partial pressure at the top of the reactor, and to use membrane separation. The method eliminates the created hydrogen, among other things, promptly and efficiently. Methanogens must accomplish the task of decreasing hydrogen partial pressure in anaerobic environments in general. However, the development and metabolism of hydrogen-producing bacteria in the hydrogen-producing fermentation system will be affected by the growth and metabolism of methane bacteria. The top gas replacement can reduce the hydrogen partial pressure inhibition in the system while exporting hydrogen, and it is suitable for engineering applications and has also been studied by some scholars and reported in the literature. Ming et al. (2002) used carbon dioxide, argon, and nitrogen to strip oxygen in the batch experimental device respectively for the hydrogen production of B49. The results showed that when nitrogen was used as the gas for stripping oxygen, the growth of the fermentative bacteria was the best, and the hydrogen production capacity was the greatest. Next is argon, and carbon dioxide is the worst as a degassing gas. Mizuno et al. (2000) stripped the hydrogen production system with 110 ml/min N_2_, which raised the hydrogen output from 0.85 mol H_2_/mol glucose to 1.43 mol H_2_/mol glucose, a 68 percent increase. Kim et al. (2006b) used a CSTR fermentation reactor for hydrogen production, and compared the effects of non-aeration, internal gas circulation ventilation, N_2_ ventilation, and CO_2_ ventilation. It was found that the effect of N_2_ and CO_2_ ventilation was better than that of non-aerated and internal gas circulation ventilation. The use of CO_2_ ventilation can inhibit hydrogen-trophic acetogenic bacteria and lactic acid bacteria, and its effect is better than that of N_2_ ventilation.

## 5 Challenge and development direction of fermentation biohydrogen production technology

Existing researches have carried out a series of researches on the control conditions of temperature, pH, substrate, residence time, gas-phase and liquid-phase products of hydrogen production by fermentation, with pure bacteria and mixed bacteria as the main body of fermentation. However, there is still room for improvement in the rate of hydrogen production, and there are still problems such as slow start-up, low system stability, and easy accumulation of volatile acids and inhibition. There are still areas for further research in hydrogen production by fermentation:

(1) There is a problem of inhibition of hydrogen partial pressure in both butyric acid and ethanol fermentation hydrogen production, and some of the hydrogen generated during the reaction will be consumed by hydrogen-consuming microorganisms. This will reduce the hydrogen production rate of the reaction system. For the anaerobic fermentation bacteria in the system, its metabolic pathway for hydrogen production by fermentation is very sensitive to changes in hydrogen partial pressure. The hydrogen partial pressure is inversely proportional to the hydrogen production, and the products of the overall metabolic pathway will change to varying degrees, which will also affect the hydrogen production process. Modern experimental methods and means should be used to study the inhibitory factor and find a way to relieve the inhibitory factor.
(2) There are great differences between different studies on the effects of different gas stripping on the hydrogen production process, and further research is needed. For example, the effect of hydrogen partial pressure on the hydrogen production process is further studied, and the gas phase control of the hydrogen production reactor process is optimized. The operation effect of the hydrogen-producing reactor directly affects the growth of hydrogen-producing bacteria and the final hydrogen-producing effect. Reducing product inhibition in the reactor is helpful for good reactor operation.
(3) From the perspective of molecular biology and microbial ecology, the entire process of continuous hydrogen production and methane production by anaerobic fermentation can be analyzed, and the reaction mechanism, organic matter degradation process, and metabolic process can be studied.
(4) Strain screening and compound flora culture. Due to the low utilization rate of organic matter in biological hydrogen production, it is necessary to carry out compound bacterial culture according to the hydrogen production capacity and metabolic level of different microorganisms to improve the substrate utilization rate and hydrogen production, and at the same time strengthen the COD removal rate of organic wastewater.
(5) Development and operation mode selection of high-efficiency anaerobic reactor. From the perspective of substrate mass transfer and microbial immobilization, research is carried out to develop a new type of anaerobic reactor that can not only efficiently degrade COD in organic wastewater, but also convert quickly and efficiently to hydrogen production, and optimize the operation mode.
(6) Development of new ways and new technologies for biological hydrogen production. The metabolic characteristics and mechanism of anaerobic microorganisms are continuously researched and explored, and new technologies for biological hydrogen production are developed by coupling electrochemistry, magnetic effect, microwave effect and other external strengthening technical means.

## 6 Summary

Biological hydrogen production technology is an ideal method to obtain clean energy hydrogen, among which anaerobic fermentation biological hydrogen production is a very promising hydrogen production technology for industrial application. The use of high-concentration organic wastewater as a fermentation substrate to conduct anaerobic fermentation biological hydrogen production research can not only produce clean and environmentally friendly renewable energy, but also reduce pollutant emissions and protect the environment, which has great research value.

In order to realize the large-scale industrial production and application of anaerobic fermentation biological hydrogen production, it is first necessary to fully understand the growth characteristics of various hydrogen-producing bacteria, and optimize the control of various influencing factors according to the ecological characteristics. Therefore, culturing and screening high-efficiency hydrogen-producing strains and optimizing reaction conditions have very important research value. This review aims to control the hydrogen production system of organic wastewater anaerobic fermentation, and to discuss the changes and effects of influencing factors on the system, as well as the impact mechanism. Taking anaerobic fermentation and biological hydrogen production by fermentation as the guiding principle, based on the process of hydrogen production by phase separation of organic wastewater fermentation, the influence of hydrogen partial pressure on the process of hydrogen production by fermentation of organic wastewater was analyzed and discussed. With the goal of strengthening the hydrogen production by wastewater fermentation, the control strategies and principles of the anaerobic fermentation hydrogen production process were investigated, so as to achieve the purpose of stable operation of the anaerobic fermentation system, intensification of the hydrogen production process, and reduction of production costs.

## References

[B1] Akhlaghi N. Najafpour-Darzi G. (2020). A comprehensive review on biological hydrogen production. Int. J. Hydrogen Energy 45, 22492–22512. 10.1016/j.ijhydene.2020.06.182

[B2] Akutsu Y. Lee D.-Y. Chi Y.-Z. Li Y.-Y. Harada H. Yu H.-Q. (2009). Thermophilic fermentative hydrogen production from starch-wastewater with bio-granules. Int. J. Hydrogen Energy 34, 5061–5071. 10.1016/j.ijhydene.2009.04.024

[B3] Ali R. Saravia F. Hille-Reichel A. Gescher J. Horn H. (2021). Propionic acid production from food waste in batch reactors: Effect of pH, types of inoculum, and thermal pre-treatment. Bioresour. Technol. 319, 124166. 10.1016/j.biortech.2020.124166 32992271

[B4] Barca C. Soric A. Ranava D. Giudici-Orticoni M.-T. Ferrasse J.-H. (2015). Anaerobic biofilm reactors for dark fermentative hydrogen production from wastewater: A review. Bioresour. Technol. 185, 386–398. 10.1016/j.biortech.2015.02.063 25746594

[B5] Behera S. Arora R. Nandhagopal N. Kumar S. (2014). Importance of chemical pretreatment for bioconversion of lignocellulosic biomass. Renew. Sustain. energy Rev. 36, 91–106. 10.1016/j.rser.2014.04.047

[B6] Chairattanamanokorn P. Penthamkeerati P. Reungsang A. Lo Y.-C. Lu W.-B. Chang J.-S. (2009). Production of biohydrogen from hydrolyzed bagasse with thermally preheated sludge. Int. J. hydrogen energy 34, 7612–7617. 10.1016/j.ijhydene.2009.07.034

[B7] Chang S. Li J. Liu F. Yu Z. (2012). Effect of different gas releasing methods on anaerobic fermentative hydrogen production in batch cultures. Front. Environ. Sci. Eng. 6, 901–906. 10.1007/s11783-012-0403-1

[B8] Chen R. Wang Y.-Z. Liao Q. Zhu X. Xu T.-F. (2013). Hydrolysates of lignocellulosic materials for biohydrogen production. BMB Rep. 46, 244–251. 10.5483/bmbrep.2013.46.5.038 23710634 PMC4133895

[B9] Chen Y. Yin Y. Wang J. (2021). Influence of butyrate on fermentative hydrogen production and microbial community analysis. Int. J. Hydrogen Energy 46, 26825–26833. 10.1016/j.ijhydene.2021.05.185

[B10] Chen Y. Zhang X. Chen Y. (2021). Propionic acid-rich fermentation (parf) production from organic wastes: A review. Bioresour. Technol. 339, 125569. 10.1016/j.biortech.2021.125569 34303105

[B11] de Menezes C. A. Silva E. L. (2019). Hydrogen production from sugarcane juice in expanded granular sludge bed reactors under mesophilic conditions: The role of homoacetogenesis and lactic acid production. Industrial Crops Prod. 138, 111586. 10.1016/j.indcrop.2019.111586

[B12] Dessì P. Asunis F. Ravishankar H. Cocco F. G. De Gioannis G. Muntoni A. (2020). Fermentative hydrogen production from cheese whey with in-line, concentration gradient-driven butyric acid extraction. Int. J. Hydrogen Energy 45, 24453–24466. 10.1016/j.ijhydene.2020.06.081

[B13] Feng X. Wang H. Wang Y. Wang X. Huang J. (2010). Biohydrogen production from apple pomace by anaerobic fermentation with river sludge. Int. J. Hydrogen Energy 35, 3058–3064. 10.1016/j.ijhydene.2009.07.015

[B14] Gadow S. I. Jiang H. Watanabe R. Li Y.-Y. (2013). Effect of temperature and temperature shock on the stability of continuous cellulosic-hydrogen fermentation. Bioresour. Technol. 142, 304–311. 10.1016/j.biortech.2013.04.102 23747441

[B15] Ghosh T. K. Prelas M. A. (2009). Energy resources and systems: Volume 1: Fundamentals and non-renewable resources. Dordrecht: Springer Netherlands.

[B16] Gray C. T. Gest H. (1965). Biological formation of molecular hydrogen: A" hydrogen valve" facilitates regulation of anaerobic energy metabolism in many microorganisms. Science 148, 186–192. 10.1126/science.148.3667.186 14259761

[B17] Guo X. M. Trably E. Latrille E. Carrere H. Steyer J.-P. (2014). Predictive and explicative models of fermentative hydrogen production from solid organic waste: Role of butyrate and lactate pathways. Int. J. hydrogen energy 39, 7476–7485. 10.1016/j.ijhydene.2013.08.079

[B18] Guochen Z. Jianzheng L. Jingbo G. Zhaohan Z. Di G. Haifeng L. (2013). Research on the operation characteristics and hydrogen production efficiency of ABR fermentation system. Chin. Environ. Sci. 33, 75–81. 10.3969/j.issn.1000-6923.2013.01.011

[B19] Hosseinzadeh A. Zhou J. L. Altaee A. Li D. (2022). Machine learning modeling and analysis of biohydrogen production from wastewater by dark fermentation process. Bioresour. Technol. 343, 126111. 10.1016/j.biortech.2021.126111 34648964

[B20] International Energy Agency (2003). Key world energy statistics. Washington, DC: IEA.

[B21] Karadag D. Köroğlu O. E. Ozkaya B. Cakmakci M. Heaven S. Banks C. (2014). A review on fermentative hydrogen production from dairy industry wastewater. J. Chem. Technol. Biotechnol. 89, 1627–1636. 10.1002/jctb.4490

[B22] Kim D.-H. Han S.-K. Kim S.-H. Shin H.-S. (2006). Effect of gas sparging on continuous fermentative hydrogen production. Int. J. Hydrogen Energy 31, 2158–2169. 10.1016/j.ijhydene.2006.02.012

[B23] Kim S.-H. Han S.-K. Shin H.-S. (2006). Effect of substrate concentration on hydrogen production and 16S rDNA-based analysis of the microbial community in a continuous fermenter. Process Biochem. 41, 199–207. 10.1016/j.procbio.2005.06.013

[B24] Kotsopoulos T. A. Zeng R. J. Angelidaki I. (2006). Biohydrogen production in granular up‐flow anaerobic sludge blanket (UASB) reactors with mixed cultures under hyper‐thermophilic temperature (70° C). Biotechnol. Bioeng. 94, 296–302. 10.1002/bit.20844 16570323

[B25] Kumar A. Samadder S. (2020). Performance evaluation of anaerobic digestion technology for energy recovery from organic fraction of municipal solid waste: A review. Energy 197, 117253. 10.1016/j.energy.2020.117253

[B26] Lee D.-Y. Li Y.-Y. Oh Y.-K. Kim M.-S. Noike T. (2009). Effect of iron concentration on continuous H2 production using membrane bioreactor. Int. J. hydrogen energy 34, 1244–1252. 10.1016/j.ijhydene.2008.11.093

[B27] Lee K.-S. Tseng T.-S. Liu Y.-W. Hsiao Y.-D. (2012). Enhancing the performance of dark fermentative hydrogen production using a reduced pressure fermentation strategy. Int. J. hydrogen energy 37, 15556–15562. 10.1016/j.ijhydene.2012.04.039

[B28] Lettinga G. Field J. Van Lier J. Zeeman G. Pol L. H. (1997). Advanced anaerobic wastewater treatment in the near future. Water Sci. Technol. 35, 5–12. 10.2166/wst.1997.0347

[B29] Levin D. B. Pitt L. Love M. (2004). Biohydrogen production: Prospects and limitations to practical application. Int. J. hydrogen energy 29, 173–185. 10.1016/s0360-3199(03)00094-6

[B30] Li J. Ai B. Ren N. (2013). Effect of initial sludge loading rate on the formation of ethanol type fermentation for hydrogen production in a continuous stirred‐tank reactor. Environ. Prog. Sustain. Energy 32, 1271–1279. 10.1002/ep.11732

[B31] Li J. Zheng G. He J. Chang S. Qin Z. (2009). Hydrogen-producing capability of anaerobic activated sludge in three types of fermentations in a continuous stirred-tank reactor. Biotechnol. Adv. 27, 573–577. 10.1016/j.biotechadv.2009.04.007 19393312

[B32] Li Z. Jiang Z. Fei B. Yu Y. (2012). Bioconversion of bamboo to bioethanol using the two-stage organosolv and alkali pretreatment. BioResources 7, 5691–5699. 10.15376/biores.7.4.5691-5699

[B33] Li Z. Qing C. Yili W. (2017). Research on rapid start-up and operation optimization of ABR for the treatment of organic matter in simulated livestock and poultry wastewater. Chin. J. Environ. Eng. 11, 3943–3951. 10.12030/j.cjee.201605034

[B34] Lin C. Lay C. (2005). A nutrient formulation for fermentative hydrogen production using anaerobic sewage sludge microflora. Int. J. hydrogen energy 30, 285–292. 10.1016/j.ijhydene.2004.03.002

[B35] Lingling G. (2014). Pretreatment of lignocellulose in water remediation plants and its anaerobic fermentation for hydrogen production. Doctoral dissertation. Zhejiang University.

[B36] Lu J. Zahedi A. Yang C. Wang M. Peng B. (2013). Building the hydrogen economy in China: Drivers, resources and technologies. Renew. Sustain. Energy Rev. 23, 543–556. 10.1016/j.rser.2013.02.042

[B37] Łukajtis R. Hołowacz I. Kucharska K. Glinka M. Rybarczyk P. Przyjazny A. (2018). Hydrogen production from biomass using dark fermentation. Renew. Sustain. Energy Rev. 91, 665–694. 10.1016/j.rser.2018.04.043

[B38] Luo G. Xie L. Zou Z. Wang W. Zhou Q. Shim H. (2010). Anaerobic treatment of cassava stillage for hydrogen and methane production in continuously stirred tank reactor (CSTR) under high organic loading rate (OLR). Int. J. hydrogen energy 35, 11733–11737. 10.1016/j.ijhydene.2010.08.033

[B39] Ma H. Zhang Y. Shen M. (2021). Application and prospect of supercapacitors in internet of energy (IOE). J. Energy Storage 44, 103299. 10.1016/j.est.2021.103299

[B40] Mengjia W. Hong S. Ruiling Z. (2014). The latest research progress of bio-fermentation hydrogen production technology. Mod. Chem. 24, 43–46. 10.3969/j.issn.0253-4320.2014.05.011

[B41] Ming L. Nanqi R. Aijie W. Fang M. (2002). High-efficiency hydrogen-producing fermentation bacteria produce hydrogen under different gas-phase conditions. China Biogas 20, 3–7. 10.3969/j.issn.1000-1166.2002.02.001

[B42] Mizuno O. Dinsdale R. Hawkes F. R. Hawkes D. L. Noike T. (2000). Enhancement of hydrogen production from glucose by nitrogen gas sparging. Bioresour. Technol. 73, 59–65. 10.1016/s0960-8524(99)00130-3

[B43] Moya D. Aldás C. López G. Kaparaju P. (2017). Municipal solid waste as a valuable renewable energy resource: A worldwide opportunity of energy recovery by using waste-to-energy technologies. Energy Procedia 134, 286–295. 10.1016/j.egypro.2017.09.618

[B44] Mu Y. Zheng X.-J. Yu H.-Q. Zhu R.-F. (2006). Biological hydrogen production by anaerobic sludge at various temperatures. Int. J. Hydrogen Energy 31, 780–785. 10.1016/j.ijhydene.2005.06.016

[B45] Muhamad N. S. Johan N. A. Isa M. H. Kutty S. R. M. (2011). “Biohydrogen production using dark and photo fermentation: A mini review,” in 2011 National Postgraduate Conference, Perak, Malaysia, 19-20 September 2011 (IEEE), 1–9.

[B46] Nanqi R. Wanqian G. Bingfeng L. (2010). Development and application prospect of biological hydrogen production technology. J. Harbin Inst. Technol. 42, 855–863. 10.11918/j.issn.0367-6234.2010.06.005

[B47] Nguyen T.-A. D. Han S. J. Kim J. P. Kim M. S. Sim S. J. (2010). Hydrogen production of the hyperthermophilic eubacterium, Thermotoga neapolitana under N2 sparging condition. Bioresour. Technol. 101, S38–S41. 10.1016/j.biortech.2009.03.041 19361983

[B48] Palmqvist E. Hahn-Hägerdal B. (2000). Fermentation of lignocellulosic hydrolysates. II: Inhibitors and mechanisms of inhibition. Bioresour. Technol. 74, 25–33. 10.1016/s0960-8524(99)00161-3

[B49] Perna V. Castelló E. Wenzel J. Zampol C. Lima D. F. Borzacconi L. (2013). Hydrogen production in an upflow anaerobic packed bed reactor used to treat cheese whey. Int. J. Hydrogen Energy 38, 54–62. 10.1016/j.ijhydene.2012.10.022

[B50] Pugazhendhi A. Kumar G. Sivagurunathan P. (2019). Microbiome involved in anaerobic hydrogen producing granules: A mini review. Biotechnol. Rep. 21, e00301. 10.1016/j.btre.2018.e00301 PMC6321885 30627520

[B51] Rambabu K. Bharath G. Thanigaivelan A. Das D. Show P. L. Banat F. (2021). Augmented biohydrogen production from rice mill wastewater through nano-metal oxides assisted dark fermentation. Bioresour. Technol. 319, 124243. 10.1016/j.biortech.2020.124243 33254466

[B52] Ren N. Li J. Li B. Wang Y. Liu S. (2006). Biohydrogen production from molasses by anaerobic fermentation with a pilot-scale bioreactor system. Int. J. Hydrogen Energy 31, 2147–2157. 10.1016/j.ijhydene.2006.02.011

[B53] Ren N. Xing D. Rittmann B. E. Zhao L. Xie T. Zhao X. (2007). Microbial community structure of ethanol type fermentation in bio‐hydrogen production. Environ. Microbiol. 9, 1112–1125. 10.1111/j.1462-2920.2006.01234.x 17472628

[B54] Reverberi A. P. Klemeš J. J. Varbanov P. S. Fabiano B. (2016). A review on hydrogen production from hydrogen sulphide by chemical and photochemical methods. J. Clean. Prod. 136, 72–80. 10.1016/j.jclepro.2016.04.139

[B55] Saady N. M. C. (2013). Homoacetogenesis during hydrogen production by mixed cultures dark fermentation: Unresolved challenge. Int. J. Hydrogen Energy 38, 13172–13191. 10.1016/j.ijhydene.2013.07.122

[B56] Shen M. Ma H. (2022). Metal-organic frameworks (MOFs) and their derivative as electrode materials for lithium-ion batteries. Coord. Chem. Rev. 470, 214715. 10.1016/j.ccr.2022.214715

[B57] Shen M. (2021). Optimization of polarization resistance of (Cu, Mn) 3O4 as cathode current‐collecting layer for solid oxide fuel cells. Adv. Energy Sustain. Res. 2, 2100110. 10.1002/aesr.202100110

[B58] Shen M. Zhang P. (2020). Progress and challenges of cathode contact layer for solid oxide fuel cell. Int. J. Hydrogen Energy 45, 33876–33894. 10.1016/j.ijhydene.2020.09.147

[B59] Sheng C. Feng L. (2016). Anaerobic acidogenic fermentation types and microbial community structure analysis of molasses wastewater at different pH. Environ. Sci. Res. 29, 1370–1377. 10.13198/j.issn.1001-6929.2016.09.16

[B60] Shima S. Pilak O. Vogt S. Schick M. Stagni M. S. Meyer-Klaucke W. (2008). The crystal structure of [Fe]-hydrogenase reveals the geometry of the active site. Science 321, 572–575. 10.1126/science.1158978 18653896

[B61] Shizas I. Bagley D. M. (2004). Experimental determination of energy content of unknown organics in municipal wastewater streams. J. Energy Eng. 130, 45–53. 10.1061/(asce)0733-9402(2004)130:2(45)

[B62] Siddiqui Z. Memon S. Brohi K. (2017). Biohydrogen production function of operating pH and seed pre-treatment. Journal-SURJ Sci. Ser. 49, 699–704. 10.26692/Surj/2017.12.43

[B63] Sivagurunathan P. Kumar G. Kim S.-H. Kobayashi T. Xu K.-Q. Guo W. (2016). Enhancement strategies for hydrogen production from wastewater: A review. Curr. Org. Chem. 20, 2744–2752. 10.2174/1385272820666160513150723

[B64] Sivaramakrishnan R. Shanmugam S. Sekar M. Mathimani T. Incharoensakdi A. Kim S.-H. (2021). Insights on biological hydrogen production routes and potential microorganisms for high hydrogen yield. Fuel 291, 120136. 10.1016/j.fuel.2021.120136

[B65] Trchounian K. Poladyan A. Trchounian A. (2017). Enhancement of *Escherichia coli* bacterial biomass and hydrogen production by some heavy metal ions and their mixtures during glycerol vs glucose fermentation at a relatively wide range of pH. Int. J. hydrogen energy 42, 6590–6597. 10.1016/j.ijhydene.2017.02.003

[B66] Usman T. M. Banu J. R. Gunasekaran M. Kumar G. (2019). Biohydrogen production from industrial wastewater: An overview. Bioresour. Technol. Rep. 7, 100287. 10.1016/j.biteb.2019.100287

[B67] Vasconcelos E. Leitão R. Santaella S. (2016). Factors that affect bacterial ecology in hydrogen-producing anaerobic reactors. Bioenergy Res. 9, 1260–1271. 10.1007/s12155-016-9753-z

[B68] Wang B. Wan W. Wang J. (2009). Effect of ammonia concentration on fermentative hydrogen production by mixed cultures. Bioresour. Technol. 100, 1211–1213. 10.1016/j.biortech.2008.08.018 18809322

[B69] Wang B. Wan W. Wang J. (2008). Inhibitory effect of ethanol, acetic acid, propionic acid and butyric acid on fermentative hydrogen production. Int. J. Hydrogen Energy 33, 7013–7019. 10.1016/j.ijhydene.2008.09.027

[B70] Wang D. Yi N. Wang Y. Yang J. Fu Q. Liu X. (2021). Triclosan degradation in sludge anaerobic fermentation and its impact on hydrogen production. Chem. Eng. J. 421, 129948. 10.1016/j.cej.2021.129948

[B71] Wang J. Wan W. (2009). Factors influencing fermentative hydrogen production: A review. Int. J. hydrogen energy 34, 799–811. 10.1016/j.ijhydene.2008.11.015

[B72] Wang Y. Xi B. Li M. Jia X. Wang X. Xu P. (2020). Hydrogen production performance from food waste using piggery anaerobic digested residues inoculum in long-term systems. Int. J. Hydrogen Energy 45, 33208–33217. 10.1016/j.ijhydene.2020.09.057

[B73] Wong Y. M. Wu T. Y. Juan J. C. (2014). A review of sustainable hydrogen production using seed sludge via dark fermentation. Renew. Sustain. Energy Rev. 34, 471–482. 10.1016/j.rser.2014.03.008

[B74] Xia Y. Ruying L. (2017). Research progress on the effects of seed mud pretreatment and fermentation temperature on hydrogen production in dark fermentation. Chin. J. Bioeng. 37, 132–140. 10.13523/j.cb.20171118

[B75] Yang G. Wang J. (2019). Changes in microbial community structure during dark fermentative hydrogen production. Int. J. Hydrogen Energy 44, 25542–25550. 10.1016/j.ijhydene.2019.08.039

[B76] Yang H. Wang X. Zhang L. Guo L. (2012). Enhanced hydrogen production performance of Rubrivivax gelatinosus M002 using mixed carbon sources. Int. J. hydrogen energy 37, 13296–13303. 10.1016/j.ijhydene.2012.06.101

[B77] Yi S. Jang Y.-C. An A. K. (2018). Potential for energy recovery and greenhouse gas reduction through waste-to-energy technologies. J. Clean. Prod. 176, 503–511. 10.1016/j.jclepro.2017.12.103

[B78] Yongfeng L. Hong C. Liran Y. Yikun C. Nanqi R. (2013). Effects of different metal ions on the hydrogen production capacity of Biohydrogenbacterium R3 sp. nov. Sol. Energy J. 34, 1280–1287. 10.3969/j.issn.0254-0096.2013.07.027

[B79] Yongfeng L. Yixuan W. Guoling C. Chunyan L. (2013). Effects of ferric ions on hydrogen production efficiency of anaerobic fermentation in UASB reactor. Environ. Sci. policy 34, 2290–2294.

[B80] Yongfeng L. Yunhan L. Qiaoyan L. Siyuan C. (2016). High-purity hydrogen production from molasses in an upflow anaerobic sludge bed reactor (UASB) at low pH. Environ. Chem. 35, 810–816. 10.7524/j.issn.0254-6108.2016.04.2015110501

[B81] Zhang J. Kan X. Shen Y. Loh K.-C. Wang C.-H. Dai Y. (2018). A hybrid biological and thermal waste-to-energy system with heat energy recovery and utilization for solid organic waste treatment. Energy 152, 214–222. 10.1016/j.energy.2018.03.143

[B82] Zhang Y. Li J. Meng J. Sun K. Yan H. (2021). A neutral red mediated electro-fermentation system of Clostridium beijerinckii for effective co-production of butanol and hydrogen. Bioresour. Technol. 332, 125097. 10.1016/j.biortech.2021.125097 33845318

[B83] Zhang Z.-P. Tay J.-H. Show K.-Y. Yan R. Liang D. T. Lee D.-J. (2007). Biohydrogen production in a granular activated carbon anaerobic fluidized bed reactor. Int. J. Hydrogen Energy 32, 185–191. 10.1016/j.ijhydene.2006.08.017

